# Obesity, Metabolic Syndrome and MASLD in Children: Inflammation as the Missing Link—A Short Narrative Review

**DOI:** 10.3390/life16020310

**Published:** 2026-02-11

**Authors:** Mihaela-Andreea Podeanu, Claudiu Marinel Ionele, Raluca Elena Sandu, Ion Rogoveanu, Mioara Desdemona Stepan, Carmen Elena Niculescu, Sergiu-Marian Cazacu, Ștefănița Bianca Vintilescu

**Affiliations:** 1 Doctoral School, University of Medicine and Pharmacy of Craiova, 200349 Craiova, Romania; mihaela.podeanu@umfcv.ro; 2 Department of Infant Care, Pediatrics and Neonatology, University of Medicine and Pharmacy of Craiova, 200349 Craiova, Romania; desdemona.stepan@umfcv.ro (M.D.S.); carmen.niculescu@umfcv.ro (C.E.N.); bianca.vintilescu@umfcv.ro (Ș.B.V.); 3 Department of Gastroenterology, University of Medicine and Pharmacy of Craiova, 200349 Craiova, Romania; ionirogoveanu@gmail.com (I.R.); sergiu.cazacu@umfcv.ro (S.-M.C.); 4 Department of Biochemistry, University of Medicine and Pharmacy of Craiova, 200349 Craiova, Romania; raluca.sandu@umfcv.ro

**Keywords:** obesity, metabolic syndrome, hepatic steatosis, MAFLD, MASLD, inflammation

## Abstract

Childhood obesity has emerged as a major global health challenge, with a marked increase in prevalence. Defined by excessive fat accumulation, it is associated with an increased risk of developing metabolic syndrome (MetS) and metabolic dysfunction-associated steatotic liver disease (MASLD). These conditions share common pathophysiological mechanisms, involving chronic low-grade inflammation, adipose tissue dysfunction, and insulin resistance. Excess weight contributes to the development of MetS even in the pediatric population through abdominal fat accumulation, dyslipidemia, hypertension, and hyperglycemia, while also creating a pro-inflammatory state that enhances hepatic fat accumulation, leading to MASLD. It is a bidirectional relationship, with MASLD increasing the risk of hypertension and the development of MetS individual components and as a whole. Adipose tissue, especially visceral fat, acts as a metabolic and immunologic organ, producing pro-inflammatory cytokines, which further accentuate insulin resistance and hepatic injury. The “three-strike” hypothesis illustrates the progression of MASLD. Several inflammatory biomarkers, including C-reactive protein, interleukins, adipokines, and serum ferritin, have been studied to monitor and predict disease progression in pediatrics. However, their diagnostic value in children remains limited due to age-related variability and lack of standardized pediatric cut-off points. A unified definition of pediatric MetS and MASLD is crucial to improve study comparability and clinical applicability. Such standardization would support the development of targeted strategies for early identification and intervention.

## 1. Introduction

In recent decades, childhood obesity has reached concerning levels worldwide, being recognized as a concerning public health issue due to its high prevalence and associated complications [1,2]. Defined as excessive fat accumulation and representing a health risk for young patients, it predisposes to multiple metabolic imbalances, leading to chronic diseases later in life. Factors such as sedentary lifestyle, dietary habits, and genetics contribute significantly to the development of this new, but old, disease [3]. Early diagnosis and management are important to prevent long-term effects that are described in the pediatric population [4].

Metabolic syndrome (MetS) is a group of interconnected metabolic imbalances including hypertension, dyslipidemia, insulin resistance, and central obesity, which is increasingly diagnosed in children, especially those with obesity [5]. It amplifies the risk of cardiovascular diseases and type 2 diabetes mellitus (T2DM), marking it as a crucial clinical entity that needs early identification and intervention [6]. The increasing prevalence of MetS is closely correlated to the rates of pediatric obesity, highlighting a direct link between these conditions and the urgency of addressing lifestyle and dietary patterns in childhood [7]. Although early diagnosis of pediatric metabolic syndrome is crucial for the prevention of long-term associated complications, there is still considerable debate and lack of consensus regarding its definition in this age group. In Table 1, we summarize the most frequently used definitions in the current literature. Although each of the existing definitions served its intended purpose at the time it was proposed, we believe that the adoption of a unified and widely applicable definition is key to addressing some of the inconsistencies and knowledge gaps that currently persist in the literature. Ideally, definitions that incorporate percentile-based criteria may offer greater accuracy by accounting for age- and sex-specific variations. However, in many countries or regions, standardized and regularly updated percentile references are lacking, which limits the applicability of such approaches in clinical and research contexts. The recent proposal by Zong et al. [8] could represent a much-needed step forward in this regard. However, while the waist-to-height ratio (WHtR) has emerged as a promising anthropometric marker in recent studies, its clinical utility in pediatric populations remains insufficiently validated. Further large-scale studies and meta-analyses are needed to clarify these issues and support the development of a consensus definition that is both practical and evidence-based.

Hepatic steatosis was initially referred to as non-alcoholic fatty liver disease (NAFLD) [9]. Over time, the association with obesity and other metabolic alterations determined researchers and clinicians to propose updated nomenclature in order to better reflect the metabolic component of the disease, leading to the introduction of the terms metabolic dysfunction-associated fatty liver disease (MAFLD) and, more recently, metabolic dysfunction-associated steatotic liver disease (MASLD) [10]. Although these entities describe largely overlapping clinical and pathophysiological conditions, they are not fully interchangeable. MAFLD is defined by the presence of hepatic steatosis in association with metabolic dysfunction, irrespective of alcohol consumption, whereas MASLD incorporates metabolic dysfunction within a broader classification of steatotic liver diseases and retains alcohol intake as a relevant discriminating factor [11]. These definitional nuances, while often subtle, may influence study populations and reported outcomes. Therefore, in the present review, the original terminology used in the cited studies is retained when reporting their findings, while MASLD is used as the reference term reflecting current consensus. MASLD ranges from simple steatosis to steatohepatitis, fibrosis, and potentially—but most rarely—cirrhosis [12,13].

Inflammation emerges as a critical pathophysiological link between obesity, MetS, and MASLD [12]. The persistent low-grade inflammatory state, mediated by cytokines, adipokines, and immune dysregulation, represents a core pathophysiological mechanism linking these entities. This highlights the role of inflammation in disease progression and supports its consideration as a strategic focus for targeted therapy [12,14].

The aim of this review is to provide an updated and integrative overview of the evidence addressing the role of inflammation in the complex interplay between obesity, MetS, and MASLD in children. Specifically, we aim to emphasize chronic low-grade inflammation as a shared feature of these conditions, to summarize some key inflammatory biomarkers and their potential clinical relevance in children, and to highlight the bidirectional relationship between MASLD and MetS. In areas where pediatric-specific evidence is limited or lacking, relevant information derived from adult studies are discussed.

**Table 1:** Definitions used for metabolic syndrome in children and adolescents.

Organization/Author	Year	Age Group	Definition	Criteria
International Diabetes Federation (IDF) [15]	2007	6–<10 years	Cannot be diagnosed in this age group.	-
		10–<16 years	o Central obesity: waist circumference ≥ 90th percentile or adult cut-off if lower; o Triglycerides ≥ 1.7 mmol/L (≥150 mg/dL); o HDL-C < 1.03 mmol/L (<40 mg/dL); o Blood pressure: systolic ≥ 130/diastolic ≥ 85 mmHg; o Fasting glucose ≥ 5.6 mmol/L (100 mg/dL) (If ≥5.6 mmol/L or known T2DM recommend an OGTT *).	Central obesity and 2 ore more other criteria
		>16 years	IDF criteria for adults.	-
Cook et al. (Modified NCEP ATP III Adult Criteria) [16]	2003	12–19 years	o Abdominal obesity: waist circumference ≥ 90th percentile for age and sex; o Triglycerides ≥ 110 mg/dL; o HDL-C ≤ 40 mg/dL (for all ages and genders); o Blood pressure ≥ 90th percentile (for age, sex and height); o Fasting glucose ≥ 110 mg/dL.	3 or more criteria
de Ferranti et al. (Modified NCEP ATP III Adult Criteria) [17]	2004	12–19 years	o Central obesity: waist circumference > 75th percentile for age and gender; o Triglycerides ≥ 1.1 mmol/L (≥100 mg/dL); o HDL-C < 1.3 mmol/L (<50 mg/dL) (boys aged 15–19 years, <1.17 mmol/L); o Blood pressure > 90th percentile for age, gender, and height; o Fasting glucose ≥ 6.1 mmol/L (≥110 mg/dL).	3 or more criteria
Cruz et al. (Modified NCEP ATP III Adult Criteria) [18]	2004	8–13 years	o Abdominal obesity: waist circumference 90th percentile (for age, gender, and Hispanic ethnicity); o Triglycerides: >90th percentile (for age and gender); o HDL-Cl < 10th percentile (for age and gender); o Blood pressure > 90th percentile (adjusted for height, age, and gender); o Impaired glucose tolerance (ADA guidelines).	3 or more criteria
Weiss et al. (Modified NCEP ATP III Adult Criteria) [19]	2004	4–20 years	o Obesity defined as BMI–Z score ≥ 2.0 (age- and sex-specific); o Triglycerides > 95th percentile (age-, sex- and race-specific); o HDL-C < 5th percentile (age-, sex- and race-specific); o Blood pressure > 95th percentile (age-, sex- and height-specific); o Impaired glucose tolerance: glucose level 140 mg/dL (7.8 mmol/L)–200 mg/dL (11.1 mmol/L) at two hours at OGTT *.	3 or more criteria
Chinese Medical Association/Chinese Pediatric Society (CPS/CMA) [20]	2012	6–<10 years	o Cannot be diagnosed in this age group.	-
		10–16 years	o Central obesity: waist circumference > 90th percentile; o Triglycerides ≥ 1.7 mmol/L; o HDL-C < 1.03 mmol/L or non-HDC-C ≥ 3.76 mmol/L; o Blood pressure > 95th percentile; o Fasting glucose ≥ 5.6 mmol/L; or 7.8 ≤ glucose < 11.1 mmol/L at OGTT or previously diagnosed T2DM.	Central obesity and 2 ore more other criteria
Ahrens et al. (IDEFICS Study) [21]	2014	2–10.9 years	o Central obesity: waist circumference ≥ 90th percentile; o Triglycerides ≥ 90th percentile; o HDL-C ≤ 10th percentile; o Blood pressure > 90th percentile; o HOMA-insulin resistance ≥ 90th percentile or fasting glucose ≥ 90th percentile.	Central obesity and 2 ore more other criteria
Zong et al. [8]	2024	6–17 years	o Waist-to-height ratio ≥ 0.50 (Europe; United States of America) or ≥ 0.46 (Asia, Africa and South America); o Triglycerides ≥ 130 mg/dL (10–17 years) or ≥ 100 mg/dL (6–9 years); o HDL-C < 40 mg/dL; o Blood pressure: 130/80 mmHg (13–17 years), or ≥ 120/80 mmHg (6–12 years); o Fasting glucose ≥ 100 mg/dL.	3 or more criteria

* Abbreviations used: IDF—International Diabetes Federation, HDL-C—High-Density Lipoprotein Cholesterol, OGTT—Oral Glucose Tolerance Test, NCEP ATP III—National Cholesterol Education Program’s Adult Treatment Panel III, ADA—American Diabetes Association, BMI—Body Mass Index, CPS/CMA—Chinese Medical Association/Chinese Pediatric Society, HOMA-insulin resistance—Homeostatic Model Assessment of Insulin Resistance.

## 2. Materials and Methods

We designed this study as a narrative review rather than a systematic one because of the heterogeneity of pediatric definitions of MetS, the evolving nomenclature of hepatic steatosis (NAFLD/MAFLD/MASLD), and the wide variability in study designs, which would have greatly limited the applicability of a systematic approach. The narrative design allowed for more flexible interpretation and easier integration of a broad range of studies. Nevertheless, this approach is inherently subject to selection and publication bias, which is acknowledged as a limitation of the present review. Accordingly, this review did not follow PRISMA guidelines, as it was not designed as a systematic review.

A comprehensive literature search was performed in the major biomedical databases such as PubMed, Scopus and Google Scholar. We tried covering publications from 2010 to 2025, without ignoring earlier studies that were relevant to the field.

The search strategy combined terms related to the area of interest, such as “childhood obesity”, “pediatric metabolic syndrome”, “MetS”, “metabolic dysfunction-associated steatotic liver disease”, “MASLD”, “non-alcoholic fatty liver disease”, “NAFLD”, “inflammation”, “low-grade inflammation”, “adipokines”, “cytokines”, and “oxidative stress”, along with inflammatory biomarkers including “C-reactive protein (CRP)”, “CRP”, “ferritin”, “interleukins”, “tumor necrosis factor-alpha (TNF-α)”, “TNF-α”, “adiponectin”, “leptin”, “resistin”, “chemerin”, and “omentin”. Additional terms related to diagnostic and therapeutic aspects—such as “insulin resistance”, “biomarkers”, “lifestyle intervention”, “antioxidants”, and “pediatric MASLD management”—were also included in the additional search. Boolean operators (“AND”, “OR”) and keyword truncations were applied to maximize the sensitivity of the search.

Titles and abstracts retrieved through the initial search were screened for relevance to the pediatric population. Full-text articles were further evaluated if they addressed epidemiology, mechanisms of inflammation, metabolic dysfunction, liver involvement or biomarkers in obesity, MetS or MASLD/NAFLD in children. Eligible studies included observational and interventional clinical studies, randomized controlled trials, longitudinal cohorts, meta-analyses, systematic reviews and narrative reviews. Only articles published in English and available in full text were considered. Studies exclusively involving adult populations, in vitro experiments without pediatric applicability, case reports, editorials without primary data and articles unavailable in full text were excluded. Reference lists of some papers were individually searched to identify additional relevant publications.

This methodology carries several limitations. Being a narrative review, it may be subject to selection and publication bias. Heterogeneity in pediatric definitions of MetS and MASLD, along with differences in biomarker assays and study populations, represents a major challenge in interpreting and comparing study results.

An additional methodological limitation relates to the evolving nomenclature of hepatic steatosis over the years (NAFLD to MAFLD to MASLD). As mentioned before, although these terms describe largely overlapping disease entities, they are based on distinct diagnostic and inclusion criteria, including differences related to metabolic dysfunction and alcohol consumption, which, generally, are not major etiopathogenic factors in pediatrics, but these definitional nuances may still influence study selection and population characterization. Therefore, to ensure conceptual accuracy and faithful reporting of results, we retained the original terminology used in the cited studies rather than applying a unified nomenclature retroactively.

Nevertheless, the structured search strategy and inclusion of recent high-quality evidence strengthen the validity of the information and provide an up-to-date overview of inflammation as the central link between pediatric obesity, MetS and MASLD.

## 3. Childhood Obesity as a Driver of Metabolic Syndrome and MASLD

### 3.1. The Global Epidemiology of Childhood Obesity and the Impact on Public Health

The latest data available (May 2025) from the World Health Organization on pediatric obesity provides concerning news about the exponential increase in cases. In 1990, according to the report, only 2% of children and adolescents aged 5–19 years old had obesity, but, by the year 2022, the number reached 8% or more than four times the initial number. It is a major health problem in high-income countries, but low- and middle-income countries also report that cases of obesity within this age group are increasing. Also, the prevalence of obesity and overweight reached 20% within this age group. When it comes to gender, there were no major differences. In addition to the many health problems arising during childhood, obesity persists most of the time into adulthood, where it is often linked to serious metabolic and cardiovascular complications. The economic impact is mesmerizing—global costs related to excessive weight are expected to reach 3 trillion dollars per year by 2030 and will be 6 times more by 2060 [22,23].

The World Obesity Federation estimated in 2019 for the year 2025 that there will be 206 million children and adolescents affected by obesity and over 254 million in 2030. According to these estimates, in 2030, in China, India, Indonesia and Brazil, there will be more than 1 million children with the same problems. According to data provided by the Centers for Disease Control and Prevention from the United States of America, in 2020, approximately 1 in 5 children were suffering due to weight gain [4,24].

A systematic review and meta-analysis published by Zhang X et al. [1] in 2024, including 2033 studies from 154 different countries and regions and involving 45,890,555 subjects, had interesting results. The prevalence of obesity varied between different countries and regions, ranging from 0.4% in Vanuatu to 28.4% in Puerto Rico; also, the highest prevalence of obesity was in Polynesia, 19.5%, and the lowest in Central Africa, 2.4%. Moreover, there was a positive association between the income, the region and the prevalence of pediatric obesity, with high-income countries showing the highest prevalence (9.3%) and low-income countries showing the lowest one (3.6%). Significant differences were observed between race and ethnicity, with the highest prevalence occurring in the Hispanic population (23.55%), compared to Asians (10.0%). When it comes to European countries, the reported rates were 3.66% in Austria, 6.29% in Bulgaria, 3.93% in France, 4.35% in Germany, 8.19% in Greece, 6.30% in Hungary, 8.49% in Italy, 6.46% in Romania, 8.21% in Serbia, 9.28% in Spain, 3.24% in Switzerland, and 7.63% in the United Kingdom.

We already mentioned that exponential increase in childhood obesity is a global health problem. Its presence has doubled or tripled in many countries because of the combined action of multiple risk factors such as urbanization, sedentary lifestyles and increased consumption of high-calorie foods. Consequences such as T2DM, cardiovascular diseases or MetS have already proven to determine high costs, and not always with good results. Likewise, the development of prevention strategies is a global emergency. Prevention using traditional strategies based on individual behavior changes have been ineffective. Current initiatives to reduce obesity focus on promoting healthier lifestyles through policies, with real changes requiring family interventions, dietary education and sustained community support. The collaboration of multidisciplinary teams appears to be a central focus, supported by consistent efforts from both the political and medical sectors [25].

### 3.2. Mechanisms by Which Obesity Promotes MetS

Obesity promotes MetS development through a set of interlinked mechanisms including insulin resistance and dyslipidemia, as well as chronic inflammation and adipokine dysregulation. According to some authors, MetS is a complication of obesity, and more clearly of central obesity [26]. The accumulation of abdominal adipose tissue directly affects the body’s response to insulin, thus contributing to the identification of patients with hyperglycemia pruning to T2DM [27,28].

Another characteristic of MetS is cardiac injury characterized by endothelial dysfunction, determined by the inability of the vessels to relax and regulate blood flow. In pediatric populations, this may clinically manifest as arterial hypertension, reflecting early metabolic and inflammatory alterations associated with excess weight. Obesity promotes the release of free fatty acids from adipose tissue, which accumulate in the liver, determining hepatic steatosis, and linking systemic metabolic dysfunction with liver involvement [29,30].

Excess adipose tissue is responsible for the increased production of inflammatory molecules such as TNF-α, interleukin-6 (IL-6), CRP and others. In this case, the endoplasmic reticulum, due to the accumulation of unfolded proteins, can trigger inflammation and contribute to metabolic dysfunction [31].

### 3.3. Obesity Is the Starting Point in the Development of Liver Fat Accumulation and Chronic Inflammation

Fatty liver disease is not a newly recognized condition, but recent studies indicate that it has become one of the most common chronic liver disorders across all age groups, affecting approximately 13% of children worldwide [32]. In the United States, its prevalence ranges between 10% and 16%, while in Asia it is somewhat lower, at around 8%. What remains consistent across regions is that the majority of cases occur in individuals with obesity, regardless of population [32,33].

Visceral adiposity, clinically objectified as high abdominal circumference, is a key determinant of hepatic alterations. Excess adipose tissue determines a large quantity of free fatty acids to be released in the blood stream. Although the liver can physiologically store and process them, when there is an overflow, lipids accumulate within hepatocytes, impairing mitochondrial function and promoting hepatic steatosis. Over time, persistent accumulation of free fatty acids leads to lipotoxicity, and the overproduction of reactive oxygen species [34], which in turn initiate an inflammatory cascade, promoting the development of steatohepatitis, which eventually progresses to liver fibrosis [35]. The progression to cirrhosis is not something we see often in the pediatric population, but there is clear evidence that it is a complication that can appear later in life [36]. Early stages of fibrosis have already been identified in children with obesity, supporting the hypothesis that it plays a central role in the start and maintenance of liver injury [37,38].

The mechanisms of the disease clearly demonstrate the role of obesity-related conditions in the development and evolution of MASLD [39]. Therefore, the treatment of MASLD must target obesity and address each associated comorbidity in an integrated manner (dyslipidemia, hyperglycemia, hypertension) [40].

The recognition of the elements initiating metabolic inflammation can contribute to the development of therapeutic targets in both prevention and treatment. The negative effects on health are devastating; the latency period, especially in children, until the establishment of diet therapy or adequate treatment contributes in direct proportion with identified biological changes. Obesity is becoming a new epidemic at a global level, so chronic inflammation has the same upward trend, with unclear perspectives on adulthood considering all the complications and components of MASLD [40,41].

## 4. Metabolic Syndrome and MASLD, a Bidirectional Relationship

MASLD is intrinsically tied to systemic metabolic imbalance, with obesity acting as the primary driver of ectopic fat deposition within the hepatic parenchyma. Since the initial conceptualization of fatty liver disease, a profound bidirectional relationship has been recognized: Excess adipose tissue triggers liver steatosis, while the resulting hepatic dysfunction further exacerbates systemic metabolic derangement [42,43]. Over time, the pathophysiological mechanisms have been clarified, and the bidirectional relationship has been preserved in the case of obesity and MASLD [38]. MASLD predisposes to the development of MetS, but MetS also exacerbates MASLD or even increases the risk of developing it in pediatric patients without a previous diagnosis in this regard [43].

Pediatric MASLD is significantly more prevalent in males than females, with studies showing rates as high as 1:2 (girls:boys). This predominance of the male gender is determined by factors such as higher visceral fat accumulation, pubertal hormonal influences, and increased metabolic risk [32,44].

The scientific literature has long documented a reciprocal relationship between fatty liver disease and MetS. As stated above, the transition to the MASLD nomenclature was strategically designed to provide a more precise and inclusive definition that directly links liver pathology to metabolic health, introducing not only negative criteria (absence of or low alcohol consumption) but also positive criteria (presence of metabolic disturbances) [45]. Data analysis conducted by the LITMUS Consortium (Liver Investigation: Testing Marker Utility in Steatohepatitis) confirmed the robustness of this change, demonstrating that 98% of the existing NAFLD patient population fits the new MASLD criteria perfectly [46,47]; still, no perfect match was found. Importantly, by preserving the term and clinical framework of steatohepatitis, now specifically Metabolic Dysfunction-Associated Steatohepatitis (MASH), the medical community has ensured the ongoing validity of legacy research. This continuity allows clinicians and researchers to generalize decades of evidence-based data to the “new” MASLD patient population with total confidence, maintaining the focus on new therapeutic measures, without the need to “restart” clinical trials [47,48].

Hypertension, commonly encountered in patients with obesity and recognized as a key component of MetS, constitutes an important cardiovascular risk factor and an independent predictor alongside MASLD. Conversely, MASLD is linked to a higher risk of hypertension development. A comprehensive bidirectional analysis of these conditions is essential to ensure the implementation of appropriate and effective preventive strategies [49]. Siafi et al. validated the hepatic steatosis index as an effective screening tool for hepatic steatosis and showed that its coexistence with arterial hypertension is associated with a higher cardiovascular risk than its association with diabetes or MetS [50]. These findings highlight the shared obesity-driven pathophysiology and the difficulty in defining clear causal relationships between them [50,51].

Insulin resistance is a central mechanism driving the bidirectional relationship between metabolic syndrome and MASLD. As a major insulin target organ, the liver is particularly susceptible to impaired insulin signaling, which, independent of obesity, promotes hepatic lipid accumulation, inflammation, and endoplasmic reticulum stress [52]. In turn, liver injury aggravates systemic insulin resistance, reinforcing this reciprocal association. Additionally, insulin resistance at the renal level contributes to vasoconstriction, sodium retention, and water reabsorption, ultimately increasing blood pressure. Together, these mechanisms illustrate the complex bidirectional interactions linking MetS components with MASLD pathophysiology [53].

## 5. Inflammation, Metabolic Disfunction and MASLD

### 5.1. What Is Chronic Low-Grade Inflammation?

Inflammation is a fundamental physiological and immunological response, that helps restore homeostasis when the human body encounters viruses, bacteria, toxins, tissue injury, or other harmful stimuli [54].

It may be classified as acute—characterized by rapid onset, intense symptoms, and short duration—chronic—develops slowly and persists for months or even years, depending on the persistence of the trigger and the organism’s capacity to resolve the process—and subacute—an intermediate form, lasting from 2 to 6 weeks [55].

This process is initially triggered locally through increased blood flow, capillary dilation, leukocyte infiltration, and the release of a variety of chemical mediators, facilitating the elimination of harmful agents and tissue repair [56].

In humans, the inflammatory system integrates both innate immunity—mediated by macrophages, dendritic cells, and mast cells that detect pathogens and activate defense responses—and adaptive immunity—involving highly specific T and B lymphocytes matured over time [57]. Cytokines are regulatory proteins produced by these cells, exerting a hormone-like effect, that act as messengers, triggering specific signals either in the same cell (endocrine) or, after being carried through the circulation, on distant target cells (paracrine) [58]. They have either pro- or anti-inflammatory properties, but rarely both, and are critical modulators of the inflammatory process and its resolution [56].

Beyond its protective role, inflammation can itself become harmful when poorly regulated or constantly activated. Likewise, chronic low-grade inflammation is defined by a persistent, subtle activation of the immune response, with elevated levels of circulating cytokines and other pro-inflammatory mediators. This status is strongly linked to the pathogenesis of several major chronic diseases, including obesity, cardiovascular disease, T2DM, gastrointestinal conditions, and certain malignancies, contributing substantially to global morbidity and mortality [54].

More precisely, low-grade chronic inflammation in childhood represents a persistent, subclinical activation of the immune system that can disrupt normal immunological maturation and function. Elevated levels of pro-inflammatory cytokines, such as TNF-α, IL-6, and interleukin-1beta (IL-1β), can alter the balance between innate and adaptive immune responses, potentially impairing the development of immunological tolerance and promoting a chronic pro-inflammatory state [59,60]. These alterations highlight the critical need for early interventions targeting modifiable inflammatory triggers in pediatric populations.

Understanding the mechanisms, triggers, and consequences of low-grade chronic inflammation is important for developing prevention and intervention strategies against the current global epidemic of chronic diseases [54].

The recent literature has introduced the concept of metaflammation in order to more accurately describe the type of chronic low-grade inflammation associated with obesity and metabolic dysfunction (such as in T2DM) [61]. Unlike the classic inflammation, previously described, which is triggered by pathogens or tissue injury and is typically acute and high-intensity, metaflammation represents a persistent, low-grade immune activation driven by nutritional overload, energy surplus, and cellular stress within metabolically active tissues such as adipose tissue, liver, pancreas and muscle [62].

Excess nutrients, particularly saturated fatty acids, activate pattern-recognition receptors such as Toll-like receptor 4 and inflammasome pathways, notably the NLR family pyrin domain containing 3 (NLRP3) inflammasome, triggering cytokine expression, oxidative stress, and mitochondrial dysfunction [63]. Although inflammasomes are essential components of innate immunity involved in pathogen defense and tissue homeostasis, their persistent activation in the setting of metabolic disturbances promotes chronic low-grade inflammation and disrupts metabolic homeostasis rather than exerting their usual protective immune function [64]. In this context, inflammasome activation interferes with insulin signaling and sustains inflammatory cascades, while mitochondrial overload and oxidative stress further amplify caspase-1-dependent cytokine release, creating a self-perpetuating cycle of insulin resistance and selective hepatic metabolic alterations. This vicious cycle favors de novo lipogenesis, dyslipidemia, hepatic steatosis, and systemic inflammatory signaling, ultimately contributing to the development of MetS [65].

In pediatric obesity, metaflammation has emerged as a key biological link between excess adiposity and the early onset of insulin resistance, dyslipidemia, and hepatic steatosis. Visceral adipose tissue becomes a central site of immune–metabolic interaction, characterized by macrophage polarization toward a pro-inflammatory phenotype, sustained cytokine production, and progressive impairment of insulin signaling pathways. These processes occur early in life, often preceding the clinical manifestation of MetS, and contribute to the progression toward MASLD. These alterations within the concept of metaflammation provide a more accurate understanding of pediatric immune–metabolic dysfunction and highlights potential targets for early prevention and therapeutic intervention [66].

### 5.2. Adipose Tissue Induced Inflammation

Chronic low-grade inflammation is considered a central mechanism in the onset and progression of hepatic steatosis in the pediatric populations and excessive adipose tissue accumulation is believed by many authors to be the common pathway linking obesity, MetS, and hepatic steatosis. Unlike acute inflammation, where the triggers are usually pathogens or trauma, in the conditions mentioned above, the adipose tissue itself becomes the main player determining a prolonged inflammatory response [67].

Adipose tissue is broadly classified based on its anatomical distribution into subcutaneous and visceral adipose tissue—surrounding organs. These differ both in origin and function, having distinct structural and metabolic roles. Visceral adipose tissue is more metabolically active, being associated with insulin resistance and cardiometabolic complications. Central/abdominal fat is more harmful when it comes to metabolic imbalance, being strongly linked to insulin resistance and metabolic dysregulations than peripheral fat deposits. This emphasizes its importance in the development of chronic low-grade inflammation and altered adipokine secretion, leading to hepatic lipid accumulation in pediatric patients [68].

During childhood and early adolescence, adipose tissue develops fast through the process called adipogenesis. Data suggests that, from birth to adolescence, there is an active generation of new adipocytes, but after, their total number remains relatively constant trough adulthood. Therefore, in adults, the increase in fat mass is mainly due to the hypertrophy of existing adipocytes, rather than the formation of new ones [69,70].

Excess adipose tissue, particularly visceral fat, is characterized by an imbalance between pro- and anti-inflammatory cytokines, with elevated production of IL-6 and TNF-α, increased oxidative stress, and higher circulating CRP levels [71].

Through the secretion of adipokines, adipose tissue modulates energy expenditure, insulin sensitivity, glucose and lipid metabolism, endothelial function, and inflammation via autocrine and paracrine mechanisms. In addition, adipose tissue stores immune cells, that function as an immune organ linking metabolism and immunity. Studies show that an increased secretion of cytokines, especially TNF-α and IL-6, which contribute to insulin resistance, is directly linked to macrophage infiltration and accumulation within adipose tissue [72,73].

The storing of fatty acids promotes oxidative processes such as lipid peroxidation, which increases reactive oxygen and nitrogen species (e.g., superoxide, nitric oxide) and amplifies oxidative stress within adipocytes. These changes further recruit peripheral immune cells into adipose tissue, sustaining a chronic inflammatory state marked by elevated TNF-α and leptin levels, and reduced anti-inflammatory factors like IL-10 and adiponectin [74]. Adipocytes may undergo further dysfunction, including endoplasmic reticulum stress, abnormal protein folding, autophagy, and eventually apoptosis [75]. Altogether, these events converge to promote inflammation of visceral adipose tissue, representing a key early step in the establishment of low-grade systemic inflammation [68]. This central role of adipose tissue inflammation highlights its importance in the pathogenesis of pediatric MASLD [73].

In response to energy imbalance (excess intake vs. insufficient use), adipocytes store excessive fatty acids, leading to hypertrophy and hyperplasia. Within visceral adipose deposits, adipocytes located farther from the vascular supply may develop hypoxia and ultimately necrosis. These necrotic adipocytes attract phagocytic immune cells, initiating a local inflammatory reaction to clear the damaged tissue [76].

### 5.3. The Three-Strike Mechanism in Pediatric MASLD

This is a conceptual framework used to describe the development and growth of pediatric MASLD, particularly in children and adolescents [77].

The first strike consists of lipid and triglyceride accumulation in hepatocytes. This primarily results from excessive caloric intake, obesity, insulin resistance, or genetic predispositions. Histologically, it is defined by the presence of intracellular triglyceride droplets within hepatocytes [78]. Clinically, hepatic steatosis develops and can be observed in ultrasound examination, but inflammation or liver damage is minimal or absent at this point and if present is reversible with proper intervention, such as lifestyle interventions [46].

The second strike, called “the phase of oxidative stress”, consist in persistent lipid accumulation leading to increased oxidative stress and mitochondrial dysfunction. Lipotoxicity occurs when excess fat accumulation within hepatocytes generates harmful intermediates like free fatty acids, reactive oxygen species (ROS) and advanced glycation end-products, causing cell stress and injury [79]. Hepatic lipid overload triggers excessive oxidant production through various ROS-generating pathways. When present in high concentrations, ROS can cause oxidative damage to cellular macromolecules, including DNA, lipids, and proteins, resulting in the accumulation of damaged molecules and subsequent liver injury [79,80]. At this stage, the liver begins to exhibit signs of cell injury and inflammation, progressing toward Non-Alcoholic Steatohepatitis (NASH), recently renamed Metabolic Dysfunction-Associated Steatohepatitis (MASH). Also, in this stage, insulin resistance and hepatocyte apoptosis can appear [81].

The third and last strike begins when chronic inflammation results in an activated immune response, recruiting immune cells such as macrophages, neutrophils, and lymphocytes. This inflammatory state promotes fibrosis through the activation of hepatic stellate cells, which leads to tissue scarring [82]. This state, if left untreated, can advance toward severe liver disease with fibrosis, cirrhosis, and potential liver failure or hepatocellular carcinoma in time [83].

The gut microbiome is considered by many an organ itself. It adapts and is reactive to every change in a child’s life, even if it is hormonal, dietary or related to something else. When dysbiosis appears, it can greatly influence the overall health status of a child, and vice versa [84].

An increasing focus on structural mechanics is recognized in the pathogenesis of pediatric MASLD is the gut–liver axis [85]. Obesity-associated intestinal dysbiosis can alter the gut barrier integrity, which may lead to an increased intestinal permeability and also to bacterial translocation, particularly lipopolysaccharides, into the portal circulation [86]; also, it is well known that intestinal permeability is higher in children patients diagnosed with steatohepatitis compared with those with steatosis only. This process boosts hepatic Toll-like receptor activation, sustained low-grade inflammation and Kupffer cell stimulation, thereby amplifying insulin resistance and hepatic lipid accumulation. These mechanisms may be particularly relevant due to the dynamic maturation of the gut microbiota and immune system during early life [87].

Extending beyond the gut–liver interface, research increasingly identifies a tripartite relation: the gut–brain–liver axis. Within this framework, microbial metabolites and inflammatory signaling modulate central appetite control and energy homeostasis [88]. Persistent activation of this axis likely sustains a pathological feedback loop, combining maladaptive eating behavior with systemic inflammation which drives the progression of obesity and MASLD [89,90]. By incorporating these microbiome-driven pathways into the traditional “three-strike” model, a more nuanced understanding of pediatric metabolic liver disease, positioning the gut microbiota as a primary site for early clinical intervention, can be approached [91,92].

Understanding this three-strike mechanism emphasizes the need for early diagnosis and targeted interventions focusing on lifestyle modifications (nutrition and physical activity) and weight management.

**Figure 1:**
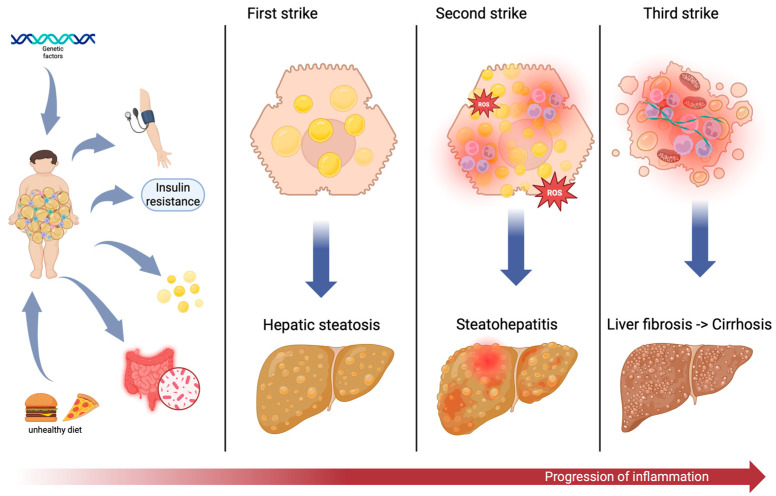
The “three-strike” hypothesis in the pathogenesis of MASLD in children. This figure illustrates the progressive pathophysiological model of MASLD, emphasizing its origins in childhood obesity. The left panel outlines key initiating factors, while in the right part is illustrated the three-strike model of MASLD progression. Created in BioRender. Podeanu, M. (2026) https://BioRender.com/zvnf4ku.

To facilitate understanding of this pathogenic sequence, Figure 1 visually illustrates the “three-strike” hypothesis, depicting how obesity-related mechanisms initiate hepatic fat accumulation, trigger inflammation, and ultimately contribute to liver fibrosis and cirrhosis.

### 5.4. Inflammatory Markers in Pediatric MASLD and MetS

In order to identify and monitor the inflammatory response, we need reliable biomarkers, which are measurable and objectively evaluable and can serve as an indicator of normal biological processes, pathogenic conditions, or pharmacological responses to interventions [93,94].

Next, we will present some of the inflammatory biomarkers used in the literature that proved to be useful in objectifying and monitoring MetS, MASLD or both in the pediatric population.

CRP is a protein produced by the liver and is one of the most cost-effective, accurate and dynamic markers and mediators of systemic inflammation. It has been determined that both CRP and highly sensitive CRP are capable of identifying inflammation in children and adolescents with MetS [95]. Also, it is associated with the severity of hepatic steatosis [96]. In a study published in 2023, it proved to be effectively used as a target for monitoring and evaluating inflammatory processes associated MASLD, especially in the context of aging. It emphasizes that elevated levels not only reflect systemic inflammation, but also directly contribute to the progression of MASLD through complementary mechanisms, thus suggesting the therapeutic potential of CRP reduction as a strategy to ameliorate liver inflammation [97].

Another useful, widely spread and accurate marker is serum ferritin, which has a dual role: It is a marker of inflammation and reflects the status of iron storage in the body. In pediatric patients, it is a good reflection of the status and severity of liver injury. Elevated ferritin levels reflect not only iron overload but also the activation of systemic inflammatory pathways, being correlated with insulin resistance, visceral obesity, and the progression of liver fibrosis in children and adolescents. Recent studies show that it may serve as a complementary non-invasive biomarker in assessing the risk of MASLD in pediatric patients with MetS. Nevertheless, it should be interpreted with caution, considering its role as an acute-phase reactant and the possible interference of chronic inflammatory syndromes [71,98].

Interleukins are a class of cytokines expressed by leukocytes and other cells throughout the body, playing a crucial role in the activation and differentiation of immune cells and also their proliferation, maturation, migration, and adhesion. They have pro-inflammatory or anti-inflammatory properties, or both [99].

The initiation and perpetuation of liver injury are facilitated by an imbalance between an excess of pro-inflammatory cytokines and an insufficiency of anti-inflammatory cytokines, leading, in time, to steatohepatitis and fibrosis. Studies suggest that the levels of pro-inflammatory interleukins, such as IL-6, IL-1β, Interleukin 8 (IL-8), and Interleukin 17 (IL-17), increase with disease severity, while anti-inflammatory interleukins, such as Interleukin 10 (IL-10), are deficient in severe stages [100].

Among the most studied interleukins in pediatric metabolic disorders, which include MetS and MASLD, is IL-6. With pleiotropic effects, IL-6 accentuates insulin resistance, maintains a state of chronic inflammation and stimulates the hepatic synthesis of acute-phase proteins (such as CRP), amplifying the inflammatory cascade. At the same time, IL-1β promotes inflammation and liver fibrosis, and causes necrosis and damage to hepatocytes, accelerating the progression from MASLD to liver fibrosis [9,101]. To support these statements, numerous clinical studies have shown increased levels of these interleukins in the serum, but also in the liver tissue of children with NASH, being associated with both the presence and severity of hepatic steatosis [9]. IL-8 is also considered a marker of progression to fibrosis. It is secreted by macrophages during cytolysis and acts as a chemotactic factor for neutrophils, thereby contributing to lobular inflammation. Moreover, its serum levels have been correlated with histological severity and progression toward NASH [26,102].

IL-17-a is a pro-inflammatory interleukin which has not received a great deal of attention in the pediatric studies—it promotes the accumulation of lipids in the hepatocytes, which makes them vulnerable to death and exacerbates the preexisting lesions. It stimulates Kupffer cells to secrete more cytokines (IL-1β, IL-6, TNF-α), reinforcing the inflammatory network that further blocks hepatic insulin signaling [103,104]. Duan Y et al. reported higher circulating levels of IL-17 in children with NAFLD compared to children with obesity without liver disease [100]. It was also reported to be a good marker for the early screening of pediatric obesity and type 1 diabetes mellitus [105].

A key factor in the amplification of hepatic inflammation is the activation of the inflammasome, particularly the NLRP3 complex, in macrophages and Kupffer cells, a process triggered by free fatty acids and lipotoxic metabolites. This mechanism converts the interleukins IL-1β and Interleukin 18 (IL-18) into their active, secretory forms. In liver diseases, the effects of IL-18 can be either pro-inflammatory or anti-inflammatory, depending on the type of stimulus, and are also associated with the metabolic syndrome. Serum IL-18 levels are positively correlated with markers of liver cytolysis, plasma triglycerides, and C-reactive protein [106]. In experimental models of NAFLD induced by a high-fat diet, IL-18-deficient mice exhibited increased body weight, alterations in carbohydrate and lipid metabolism, with the consequent development of insulin resistance and steatosis, compared with wild-type control animals. These metabolic alterations observed in IL-18 knockout mice subjected to a high-fat diet were reversible by the administration of recombinant IL-18 protein, demonstrating a possible protective role of IL-18 in NAFLD [107].

The main anti-inflammatory cytokine, with a protective role against fat accumulation in hepatocytes demonstrated in animal studies, is represented by IL-10. Its role is to suppress excessive activation of immune cells and to protect tissues from inflammatory damage. In the liver, it is expressed by hepatocytes, Kupffer cells and stellate cells; here, it attenuates the release of pro-inflammatory cytokines and stimulates the processes of liver repair and regeneration after injury [108]. A study in obese children did not find a clear relationship between serum IL-10 and the presence of metabolic syndrome or NAFLD. However, an insufficient IL-10 response (or a decrease in the IL-10/pro-inflammatory cytokine ratio) in steatosis liver may lead to lack of control of inflammation and more extensive liver damage. Therefore, IL-10 can be viewed as a “brake” on liver inflammation: Its presence in adequate amounts limits damage, while its absence or diminution allows pathogenic cytokines (IL-1β, IL-6, TNF-α, IL-17, etc.) to cause severe steatohepatitis [109].

TNF-α—a protein mapped on chromosome 6—is mainly produced by activated macrophages, T lymphocytes, and natural killer cells [110]. It is an overexpressed pro-inflammatory cytokine contributing to insulin resistance by interfering with insulin receptor signaling, in the pathogenesis of pediatric MASLD. TNF-α initiates the “second strike”, promoting its progression towards inflammatory and fibrotic lesions. In a recent study on pediatric patients, TNF-α levels were higher in NAFLD subjects and positively associated with the severity of liver destruction [111].

Adiponectin is a hormone with anti-inflammatory and insulin sensitization properties, which protects against fat accumulation in the liver. In obesity and pediatric NAFLD, it is markedly suppressed, with children with NAFLD having significantly lower serum adiponectin levels compared to normal-weight peers. The decrease in adiponectin occurs early, even before the increase in pro-inflammatory cytokines, and is inversely correlated with BMI, insulin resistance, and liver enzymes levels. By reducing its protective effects, adiponectin deficiency favors fat deposition in hepatocytes and worsens the inflammation. A study addressing children, in which liver biopsy was performed, showed that reduced adiponectin is associated with an increased likelihood of disease, and has diagnostic value [112,113].

In the context of pediatric hepatic pathology, leptin is a hormone traditionally recognized for managing satiety and metabolic homeostasis which undergoes a deleterious functional shift. In children with NAFLD, leptin transitions from a metabolic regulator into a pro-inflammatory and profibrogenic agent, actively driving the progression of liver damage [114]. Clinical data demonstrates that pediatric NAFLD patients exhibit systemic leptin elevations that significantly exceed those of healthy controls; notably, this increase persists even when statistically controlling for BMI [115]. This suggests that the hormone’s elevation is a direct consequence of the disease state rather than merely an artifact of adipose volume [116]. Furthermore, the correlation between leptin levels and the transition from simple steatosis to NASH underscores its involvement in active tissue inflammation [117]. While its strong correlation with general obesity currently obscures its specificity as a standalone diagnostic biomarker, the leptin signaling pathway remains a high-priority target for future anti-fibrotic therapies aimed at halting liver scarring in younger populations [114].

Resistin acts as a potent pro-inflammatory mediator, primarily synthesized by macrophages within fat tissue. It is a central driver in the onset of systemic insulin resistance and the maintenance of chronic, low-grade inflammation. By destabilizing glucose regulation and encouraging inflammatory cells (macrophages) to infiltrate the liver, resistin significantly worsens hepatic injury [118]. However, clinical observations in children with obesity reveal a biological paradox: Rather than increasing with disease severity, resistin concentrations actually decline as liver steatosis becomes more advanced [119]. Data shows a clear negative correlation where children with severe fatty liver exhibit lower serum resistin levels than those with mild cases. This counterintuitive finding suggests two primary possibilities: Either the fat tissue’s secretory capacity has reached a state of “exhaustion” due to relentless inflammation, or the pediatric body is attempting a complex maladaptive adjustment to long-term metabolic stress [120]. This unexpected result could indicate that secretion is exhausted by persistent inflammation or that the body adapts to long-term inflammation.

Chemerin is a relatively recently characterized adipokine, primarily synthesized by adipose and hepatic tissues, with established functions in immune chemotaxis and the differentiation of adipogenesis. In pediatric populations, chemerin serves as a pivotal molecular bridge connecting systemic obesity to localized hepatic inflammation [121]. Elevated systemic concentrations of chemerin facilitate the pathological recruitment of macrophages and natural killer (NK) cells into the liver parenchyma, a process that exacerbates insulin resistance and accelerates steatosis. Functionally, chemerin operates as a metabolic messenger that alerts the liver to peripheral energy surpluses, thereby translating visceral adiposity into hepatic inflammation [122]. In children with obesity and hepatic steatosis, chemerin levels were elevated, which has been correlated with both the presence and severity of fat accumulation [123]. Some studies have determined that it is one of the most promising markers for assessing hepatic fat storage in the pediatric age and can possibly be a novel therapeutic target. Strategies aimed at reducing chemerin levels through lifestyle modification or pharmacological intervention could attenuate metabolic inflammation. However, further research is needed to determine whether modulating the chemerin pathway can positively impact clinical outcomes [124].

Omentin-1 is a potent anti-inflammatory adipokine synthesized primarily by the vascular stromal cells within visceral fat. Unlike pro-inflammatory markers, omentin-1 is metabolically beneficial, enhancing insulin sensitivity and providing a protective buffer against metabolic dysfunction and atherosclerosis. While adult obesity is typically characterized by a pathological reduction in omentin-1, its behavior in pediatric populations suggests a more nuanced role [116]. In children with obesity, declining omentin-1 levels are associated with worsening insulin resistance. However, data concerning pediatric NAFLD remains contradictory, likely due to the confounding influences of pubertal development and complex regulatory loops. Specifically, research from a Turkish cohort of 49 children revealed a “compensatory paradox”: Omentin-1 levels were significantly higher in children with ultrasound-proven fatty liver than in those without. Furthermore, these levels appeared to climb in direct proportion to the severity of the fat accumulation (Grade 3 > Grade 2 > Grade 1). This suggests that, in the early stages of pediatric liver stress, the body may trigger a compensatory surge of omentin-1 to counteract the burgeoning inflammation, or that the hormone is uniquely modulated by the endocrine shifts in puberty [116,125].

Conversely, a different clinical study involving 88 adolescents (56 with obesity and 32 controls) observed that serum omentin-1 was significantly suppressed in all participants with obesity, regardless of their liver status. Notably, within the NAFLD cohort, lower omentin concentrations were strongly associated with higher levels of liver enzymes like alanine aminotransferase (ALT), aspartate aminotransferase (AST), HOMA-IR, and insulin in young adults [126]. This suggests that omentin-1 is not actually a diagnostic marker, but one of metabolic dysregulation. While its clinical use as a biomarker is currently hindered by high variability, its insulin-sensitizing effects make it a prime therapeutic candidate [116,126]. Modulating omentin through high-fiber nutrition, exercise, or synthetic analogs could potentially reverse liver fat accumulation in children. Further studies are needed to clarify its mechanistic role and therapeutic applicability [127].

Recently, researchers have shifted their attention to finding more accessible and cost-effective markers to highlight the undeniable low-grade inflammation in MetS and hepatic steatosis, that can be used in a daily clinical context.

Due to the cost and complexity of measuring specialized adipokines, clinical attention has pivoted toward complete blood count (CBC)-derived inflammatory indices. These accessible, cost-effective markers—including the neutrophil-to-lymphocyte ratio (NLR), platelet-to-lymphocyte ratio (PLR), and the Systemic Immune–Inflammation Index (SII)—serve as proxies for the body’s immune balance [128]. By calculating the ratio of pro-inflammatory cells (neutrophils and monocytes) against anti-inflammatory cells (lymphocytes), clinicians can quantify the degree of low-grade inflammation associated with MetS. These metrics are highly valuable as non-invasive screening and prognostic tools in pediatric MASLD [129]. In a previous retrospective study we examined the utility of various paraclinical inflammatory and cardioembolic indexes in distinguishing between children with and without MetS and determined that subjects with MetS exhibited significantly higher values of markers such as TG/HDL-C (triglycerides/HDL-C), TC/HDL-C (total cholesterol/HDL-C), monocyte-to-HDL-C ratio (MHR), lymphocyte-to-HDL-C ratio (LHR), neutrophil-to-HDL-C ratio (NHR), atherogenic index of plasma, and platelets-to-HDL-C ratio (PHR) [130]. Thus, we decided these markers are worth describing further.

NLR is defined by dividing the absolute neutrophil count by the lymphocyte count, which serves as a streamlined metric for assessing subclinical systemic inflammation and frequently mirrors CRP levels [131,132]. In adult populations, a high NLR is a robust indicator of advanced liver fibrosis and poor clinical outcomes, with elevated values often predicting the presence of a fatty liver [133]. However, this correlation does not translate seamlessly to pediatric patients. In children, research has failed to establish a definitive threshold that distinguishes patients with obesity and with liver disease from those without it [128]. This suggests that, while NLR is a reliable inflammatory signal in adults, it is not yet ready to act as a diagnostic tool for MASLD in the younger population, likely due to the different immune profiles and developmental stages of children [134].

PLR is a composite hematological metric that assesses the interplay between systemic inflammation and prothrombotic (clot-promoting) activity. Biologically, a high PLR driven by an increase in platelets alongside a reduction in lymphocytes indicates an aggressive inflammatory state characterized by heightened platelet aggregation and a weakened lymphocyte-mediated immune response [135]. In adult populations with NAFLD, recent meta-analyses have uncovered a counterintuitive trend: PLR values are often slightly lower than in healthy controls. This paradox likely reflects the complex regulatory shifts that occur during chronic metabolic stress, such as a relative increase in lymphocytes or a decline in the reactivity of megakaryocytes as the disease enters advanced stages [133]. In pediatric populations, available data on PLR is limited; one study found no significant association between PLR and NAFLD in obese children, suggesting limited standalone diagnostic utility [128,136]. However, PLR is part of the broader panel of inflammatory indices considered in assessing low-grade inflammation in childhood obesity and may contribute to cardiometabolic risk prediction in multivariate models [137].

Conversely, MHR is an emerging composite marker integrating the inflammatory component (circulating monocytes, precursors of hepatic macrophages) with the metabolic profile (HDL cholesterol, typically reduced in metabolic syndrome). In obese children, MHR has been significantly higher in those with hepatic steatosis compared to those without [129,138]. One study on 504 children reported a statistically significant difference in MHR in group comparison [139]. Multivariate analysis identified elevated MHR as an independent predictor of pediatric NAFLD, associated with a modest but significant increase in risk after adjusting for age, waist circumference, and ALT. Further, MHR positively correlated with insulin resistance, hypertriglyceridemia and hepatic fat accumulation, indicating its relevance in metabolic dysfunction [138]. A cut-off around 0.43 discriminated children with MAFLD with ~75% sensitivity, and MHR in the highest quartile was associated with over a 10-fold increase in hepatic injury odds in some analyses. Elevated MHR reflects a combination of pro-inflammatory monocytosis and low HDL levels, which promotes hepatic and vascular inflammation. Together, current pediatric studies suggest MHR could complement traditional screening markers, although validation in larger cohorts and against quantitative imaging/biopsy endpoints remains imperative [138,139].

For ease of reference, Table 2 provides a concise summary of the main biomarkers discussed in this review.

## 6. Conclusions

Childhood obesity represents a critical starting point for the development of metabolic dysfunctions, including MetS and MASLD, which together pose an escalating threat to pediatric health worldwide. This review highlights how inflammatory processes triggered by excess adipose tissue play a central role in the pathogenesis of both MetS and MASLD in children.

From a clinical perspective, the evidence presented shows the importance of cautious interpretation of inflammatory biomarkers in pediatric populations. Although several markers correlate with disease severity and progression, their clinical applicability remains limited due to age-related variability and the absence of standardized pediatric cut-off points. Currently, these biomarkers should be viewed as supportive tools for risk stratification, rather than as standalone diagnostic indicators.

To bridge the existing gaps in the literature and move beyond the use of inflammatory markers as only research instruments, a unified definition of pediatric MetS and MASLD is essential. This standardization will allow the development of more consistent studies and ultimately support meaningful clinical progress in this emerging field.

## Figures and Tables

**Table 2:** Summary of key inflammatory biomarkers discussed in relation to pediatric MetS and MASLD.

Biomarker	Biological Role in Inflammation	Association with MetS	Association with MASLD	Main Limitations/Confounders
CRP	Acute-phase reactant indicating systemic low-grade inflammation	Strongly associated; correlates with insulin resistance and obesity	Associated with steatosis severity	Not specific; influenced by infections and adiposity
Ferritin	Iron storage protein and acute-phase reactant	Correlates with visceral obesity and insulin resistance	Associated with hepatic injury and fibrosis progression	Affected by iron status and systemic inflammation
IL-6	Pro-inflammatory cytokine; induces hepatic CRP synthesis	Elevated in pediatric MetS; linked to arterial stiffness	Correlates with disease severity and fibrosis progression	Not routinely used in clinical settings; lacks specificity
IL-1β	Pro-inflammatory cytokine involved in metabolic inflammation	Correlated with obesity, insulin resistance, and dyslipidemia	Associated with liver fibrosis	Limited clinical use; nonspecific
IL-8	Pro-inflammatory chemokine; attracts neutrophils	Associated with adipose tissue dysfunction in obese children	Marker of fibrosis progression	Primarily used in acute conditions; nonspecific
IL-17	Pro-inflammatory interleukin promoting immune-mediated inflammation	Useful marker in obesity and type 1 diabetes screening	Promotes hepatic lipid accumulation	Not disease-specific; limited availability
TNF-α	Central pro-inflammatory cytokine; promotes insulin resistance	Elevated in obesity-related MetS; key in metabolic inflammation	Associated with steatohepatitis development	Not specific; limited use in routine clinical care
Adiponectin	Anti-inflammatory adipokine; improves insulin sensitivity	Reduced levels in MetS	Inversely correlated with steatosis; potential diagnostic marker	Influenced by adiposity and puberty
Leptin	Appetite-regulating hormone; becomes pro-inflammatory at high levels	Elevated in obesity	Associated with disease progression	Strongly influenced by overall adiposity
Chemerin	Adipokine involved in chemotaxis and adipogenesis; modulates inflammation	Elevated in obesity and insulin resistance	Correlates with hepatic steatosis and fibrosis	Emerging marker; lacks pediatric reference ranges
Resistin	Pro-inflammatory adipokine linked to insulin resistance and oxidative stress	Elevated in children with MetS	Associated with steatosis and hepatic inflammation	Limited pediatric data; influenced by obesity
Omentin-1	Anti-inflammatory adipokine; improves insulin sensitivity	Decreased in children with obesity and MetS	Inversely correlated with MASLD severity	Affected by pubertal status; not routinely measured
NLR	Neutrophil-to-lymphocyte ratio; reflects systemic inflammation	Elevated in MetS-related inflammation	Inconsistently associated with MASLD in children	Age- and infection-dependent; requires cautious interpretation
PLR	Platelet-to-lymphocyte ratio; marker of inflammation and thrombocytic activity	Associated with cardiometabolic risk	Limited and inconsistent data	Influenced by infections and platelet variability
MHR	Monocyte count relative to HDL-C; reflects monocyte-driven inflammation	Elevated in MetS	Correlates with hepatic steatosis	Influenced by infections and lipid profile
TG/HDL-C ratio	Surrogate marker of insulin resistance	Strongly associated with MetS	Indirectly associated via metabolic dysfunction	Not specific; affected by fasting state and pubertal changes

## Data Availability

No new data were created or analyzed in this study.

## Abbreviations

The following abbreviations are used in this manuscript:

MetS	Metabolic syndrome
T2DM	Type 2 diabetes mellitus
NAFLD	Non-alcoholic fatty liver disease
MAFLD	Metabolic dysfunction-associated fatty liver disease
MASLD	Metabolic-associated steatotic liver disease
IDF	International Diabetes Federation
HDL-C	High-Density Lipoprotein Cholesterol
OGTT	Oral Glucose Tolerance Test
NCEP ATP III	National Cholesterol Education Program’s Adult Treatment Panel III
ADA	American Diabetes Association
BMI	Body Mass Index
CPS/CMA	Chinese Medical Association/Chinese Pediatric Society
HOMA-IR	Homeostatic Model Assessment of Insulin Resistance
CRP	C-reactive protein
TNF-α	Tumor necrosis factor-alpha
IL-6	Interleukin-6
LITMUS	Liver Investigation: Testing Marker Utility in Steatohepatitis
IL-1β	Interleukin-1beta
NLRP3	NLR family pyrin domain containing 3
ROS	Reactive oxygen species
NASH	Non-Alcoholic Steatohepatitis
IL-8	Interleukin 8
IL-17	Interleukin 17
IL-10	Interleukin 10
IL-18	Interleukin-18
NK	Natural killer cell
ALT	Alanine aminotransferase
AST	Aspartate aminotransferase
CBC	Complete blood count
NLR	Neutrophil-to-lymphocyte ratio
PLR	Platelet-to-lymphocyte ratio
SII	Systemic Immune–Inflammation Index
TG/HDL-C	Triglycerides/HDL-C
TC/HDL-C	Total cholesterol/HDL-C
MHR	Monocyte-to-HDL-C ratio
LHR	Lymphocyte-to-HDL-C ratio
NHR	Neutrophil-to-HDL-C ratio
PHR	Platelets-to-HDL-C ratio
